# From Prediabetes to Type 2 Diabetes Mellitus in Women with Polycystic Ovary Syndrome: Lifestyle and Pharmacological Management

**DOI:** 10.1155/2020/6276187

**Published:** 2020-06-08

**Authors:** Arianna Pani, Ilaria Gironi, Giacoma Di Vieste, Elena Mion, Federico Bertuzzi, Basilio Pintaudi

**Affiliations:** ^1^Postgraduate School of Clinical Pharmacology and Toxicology, University of Milan, Milan, Italy; ^2^Diabetes Consultant, Milan, Italy; ^3^Diabetes Unit, Cantù Hospital, Abbiategrasso, Italy; ^4^Diabetes Unit, Niguarda Cà Granda Hospital, Milan, Italy

## Abstract

**Aims:**

Despite the very clear association between polycystic ovary syndrome (PCOS) and dysglycemia, few studies have explored the continuum of glycemic alterations leading from minor glucose abnormalities to overt diabetes. The purpose of this review is to trace the natural history of glycemic alteration in women with PCOS.

**Methods:**

We performed a literature review without time limit until August 2019. Inclusion criteria were studies addressing the association between impaired glucose tolerance or impaired fasting glucose or type 2 diabetes (T2D) and PCOS with at least an English abstract. The exclusion criteria were no PCOS or impaired glucose tolerance or impaired fasting glucose or T2D as outcome. The outcomes of interest were the onset of impaired glucose tolerance, impaired fasting glucose, T2D, and the progression from impaired glucose tolerance or impaired fasting glucose to T2D.

**Results:**

Healthy diet and physical activity are the first-line therapy for PCOS. Treatment with metformin was associated with significant lower 2-hour postload glucose levels and with reduction in fasting glucose when compared to placebo. Thiazolidinediones were more effective in reducing fasting glucose levels compared to placebo. Metformin and pioglitazone treatments showed similar effects on fasting glucose levels. The sodium-glucose cotransporter-2 inhibitor empagliflozin did not show differences in metabolic parameters when compared to metformin. The combination therapy with metformin plus the glucagon-like peptide-1 receptor agonist liraglutide was associated with significant improvements in basal and postload glucose levels compared with only liraglutide. Likewise, a combination therapy with the dipeptidyl peptidase-4 inhibitor saxagliptin and metformin demonstrated superiority versus metformin in fasting glucose and oral glucose tolerance test normalization. Myo-inositol supplementation was associated with lower insulin levels, glucose levels, and insulin resistance when compared with placebo, metformin, or estrogen treatments.

**Conclusions:**

The use of insulin-sensitizing agents, such as metformin and inositols, along with lifestyle interventions may improve the metabolic profile in PCOS women.

## 1. Introduction

Diabetes mellitus is a worldwide epidemic. Its prevalence and incidence are steeply growing with an estimated 425 million of people currently having diabetes [1]. The great social burden of the disease is worsened by the huge number of people with prediabetes (i.e., impaired fasting glucose and/or impaired glucose tolerance) who are at high risk of developing it. In addition, one in two adults with diabetes (about 212 million of people) is undiagnosed. Several risk factors for the development of the disease have been well recognized. Some risk factors are gender specific, such as gestational diabetes mellitus (GDM) and polycystic ovary syndrome (PCOS). PCOS is defined by its reproductive features of hyperandrogenism, chronic oligoanovulation, and/or polycystic ovarian morphology [2]. Its prevalence is 5–15%, depending on the diagnostic criteria applied [3]. PCOS is associated with metabolic abnormalities, including insulin resistance (IR) and *β*-cell dysfunction [2]. The result of IR is hyperinsulinemia, which has a central role in the pathogenesis of androgen excess in PCOS. Indeed, insulin acts as a cogonadotropin to increase luteinizing hormone (LH)-induced androgen synthesis in theca cells [4] and can enhance gonadotropin-releasing hormone (GnRH)-mediated gonadotropin secretion [5]. Insulin also reduces hepatic sex hormone binding globulin (SHBG) synthesis, thereby increasing the levels of bioavailable androgens [6].

The defect of insulin action was quantified in PCOS using the euglycemic clamp [7]. Insulin action was reduced by 35–40% in both lean and obese women with PCOS compared to control women of similar age and body composition [8].

Recent studies in daughters of women affected by PCOS have found evidence for pancreatic *β*-cell dysfunction prior to menarche [9]. Genetic analyses showed that metabolic abnormalities such as obesity and IR contribute to the pathogenesis of PCOS [10]. Women with PCOS have a higher cardiometabolic risk compared with women without ovarian problems [11].

In women with PCOS, dysglycemia typically consists of impaired glucose tolerance [12], its prevalence being of almost 30% in both adult women [13] and affected adolescents [14]. For this reason, PCOS is associated with a two times increased risk for type 2 diabetes (T2D) [15].

Despite the very clear association between PCOS and dysglycemia, few studies have explored the continuum of glycemic alterations leading from minor glucose abnormalities to overt diabetes. The purpose of this review is to summarize the effect of lifestyle and pharmacological management on glycemic alterations of women with PCOS.

## 2. Materials and Methods

We searched the online databases of PubMed/Medline, Scopus, Web of Science, Science Direct, Embase, CINAHL, EBSCO, and Google Scholar search engine using MeSH keywords of “impaired glucose tolerance” or “IGT,” “impaired fasting glucose” or “IFG,” “type 2 diabetes” or “T2D,” and “polycystic ovary syndrome” or “PCOS” or “PCO syndrome” without time limit until August 2019. All the reference lists of the retrieved articles were reviewed.

All steps of the study were independently performed by two researchers and any disagreement between researchers was resolved by a third researcher.

Inclusion criteria were studies addressing the association between impaired glucose tolerance or impaired fasting glucose or T2D and PCOS with at least an English abstract with a special focus on the available PCOS treatment.

The exclusion criteria were as follows: no PCOS or impaired glucose tolerance or impaired fasting glucose or T2D as outcome, conference presentations, and letters to the editors.

The outcomes of interest were the onset of impaired glucose tolerance, impaired fasting glucose, T2D, and the progression from impaired glucose tolerance or impaired fasting glucose to T2D with a special focus on the available PCOS treatment.

## 3. Results and Discussion

### 3.1. Pathogenesis of Prediabetes and Type 2 Diabetes

Several risk factors have been hypothesized having a causal role in the pathogenesis of prediabetes and T2D of women with PCOS. Classical risk factors such as genetic background, obesity, and familiarity for diabetes and PCOS-specific risk factors have been described. The role of obesity does not impact independently on the onset of prediabetes and T2D because it was shown that also lean women with PCOS have a high risk of glucose alterations. However, obesity surely represents a strong additional risk factor. Among the PCOS-specific factors, IR and hyperandrogenism have been reported. Subjects with hyperandrogenism have a higher level of IR compared with those without hyperandrogenism [16]. Treatment with antiandrogens improves insulin sensitivity; therefore hyperandrogenism could contribute to the pathogenesis of prediabetes and T2D through mechanism of sustaining higher level of IR. However, hyperandrogenism in PCOS could worsen glucose tolerance by stimulating low-grade inflammation [17, 18].

Even if IR is not a feature always present in patients with PCOS, it is considered one of the key elements underlying the pathogenesis of the syndrome. It is the main factor associated with the development of T2D in those women. Reported prevalence of IR in women with PCOS varies from 44% to 70% [19] mostly because of different methods used to assess it [20]. IR is the typical condition of subjects with T2D. Women with PCOS share with people with T2D the same impaired glucose pattern consisting of a prevalent disturbance of fasting blood glucose. Higher levels of IR stress the pancreatic beta cell function, resulting in earlier functional depletion of insulin secretion capacity and higher risk of developing prediabetes and T2D. The other key element characterizing PCOS is hyperandrogenism.

The relation between hyperandrogenism and IR is a chicken and egg problem. On one side, androgens may have a direct role in the inhibition of hepatic and peripheral insulin action by reducing amount and efficiency of the glucose transporter type 4 (GLUT4), especially in adipose tissue and muscles [21]. Moreover, androgens in association with increased free fatty acids (FFA) levels (a common feature in women with PCOS) reduce insulin excretion in the liver and insulin-dependent glucose uptake in skeletal muscles contributing to IR and compensatory hyperinsulinemia [22]. On the other side, it is well demonstrated that insulin has a direct role in ovarian steroidogenesis and in the ovulation control. Insulin in fact directly stimulates androgen production from ovaries, enhancing the activity of CYP17*α* and other steroidogenic enzymes with increased androgen production. It also inhibits the hepatic synthesis of SHBG and the secretion of the insulin-like growth factor-binding protein (IGFBP-1) resulting in elevated levels of free IGF and androgens. Furthermore, at pituitary level, insulin stimulates LH secretion, which, in association with insulin itself, acts synergistically on theca cells, increasing androgen biosynthesis [4, 23].

As shown by Dunaif et al., IR associated with PCOS, seems to be determined also by alterations at the level of the insulin receptor signalling. They observed that insulin receptors isolated from fibroblast cultured and skeletal muscle of women with PCOS presented increased insulin-independent autophosphorylation which is associated with reduced receptor activity [24, 25] and can lead to IR. However, other abnormalities have been observed also in downstream pathways, such as the serine phosphorylation of the insulin receptor substrates which inhibits its binding with PI3K and prevents the propagation of the signal downstream [26, 27]. Because of this central role of IR and compensatory hyperinsulinemia in the pathogenesis of the disease, it appears clear how insulin sensitizers represent possible therapeutic agents.

Alternative mechanisms of the pathogenesis of prediabetes and T2D in subjects with PCOS are muscle mitochondrial dysfunction [28] and the gut microbiome [29]. The latter hypothesis is sustained by the presence of specific taxa of gut microorganisms that are associated with lower androgen levels.

The following sections and Table 1 report existing evidence of the effect of different treatments on impaired glucose tolerance and impaired fasting glucose in women with PCOS. No treatment was effective in restoring normal glucose tolerance or reverse to impaired glucose tolerance or to impaired fasting glucose when T2D was already diagnosed.

### 3.2. Lifestyle Modifications

Obesity and abdominal obesity have a significant impact on psychosocial status, quality of life, hyperandrogenism, IR, lipid profiles, ovulation, menstrual cycle, fertility, risks for T2D, and cardiovascular disease onset [30, 31]. It also may increase pregnancy complications and the risk of preeclampsia. It seems that women suffering from PCOS, with or without resistance to insulin, have a lower basal metabolic rate (BMR) compared to healthy women. In fact, they often complain about the lack of, or slow rate of, weight loss in spite of a low-calorie diet [32].

Lifestyle modification is the first-line therapy for women with PCOS [33]. It includes both diet and physical activity which are suggested to delay the onset of T2D.

Appetite regulation is a process that involves the endocrine system and the central nervous system, and its alteration is observed among these women, causing some problems with weight control. Levels of ghrelin and cholecystokinin—hormones which play essential roles in regulating appetite—are also impaired in these patients. The International Evidence-based Guideline for the Assessment and Management of Polycystic Ovary Syndrome 2018 recommended healthy lifestyle behaviours for all women with PCOS regardless of presenting symptoms [34]. Particularly for overweight women with PCOS, lowering body mass by 5% can reduce serum insulin and testosterone levels and improve menstrual and reproductive function [35].

A variety of dietary approaches should be taken into consideration. A negative energy balance of approximately 30% to achieve an energy deficit of 500–750 kcal per day is suggested to achieve weight loss through diet modification in women with PCOS [34]. These recommendations are consistent with general population guidelines suggesting low-calorie diets with sufficient carbohydrates and proteins with low-fat intake. Diet advice often requires a reduction in dietary glycemic index in addition to overall calorie reduction to induce weight loss [36].

Low glycemic index diet has metabolic effect consisting in reducing triglycerides and free fatty acids, glucose uptake, and improving insulin sensitivity. Also a low-carbohydrate ketogenic diet could be beneficial in improving insulin sensitivity [37]. However, all diets that aim to reduce weight are considered beneficial [29].

Physical activity (i.e., any bodily movement produced by skeletal muscles that requires energy expenditure) and exercise (i.e., activity requiring physical effort, carried out to sustain or improve health and fitness) improve metabolic status of women with PCOS.

The international evidence-based guideline for the assessment and management of polycystic ovary syndrome (2018) recommended that different durations and intensities of exercise are suggested for different age groups in order to prevent weight gain and maintain health.

Adults aged 18–64 should perform physical activity for a minimum of 150 min per week of moderate intensity, 75 min per week of vigorous intensity, or an equivalent of both. Recommendations for adults include muscle-strengthening activity on two nonconsecutive days. For adolescents, it is recommended for a minimum of 60 min per day most days of the week. The physical activity should be of moderate to vigorous intensity and complemented with muscle-strengthening activities performed at least three times per week.

High intensity aerobic exercise with heart rate >80% improves IR in women with PCOS [38]. However, both high intensity interval training and strength training improve IR and body composition in women with PCOS [39].

The International Evidence-based Guideline for the Assessment and Management of Polycystic Ovary Syndrome 2018 recommended the inclusion of behavioural strategies in lifestyle interventions to optimise weight management, improve general health, and maintain emotional wellbeing in PCOS women [21]. Behavioural therapy involves implementing goal setting, self-monitoring, identifying barriers, and problem solving. These strategies increase program adherence and efficacy [40].

### 3.3. Metformin

Metformin exerts its hypoglycemic action mainly at the liver, intestine, skeletal muscle, endothelium, adipose tissue, and ovary level. One of its most known effects occurs at the mitochondrial level in the liver where metformin inhibits the Complex 1 of the respiratory chain, suppressing adenosine 5′-triphosphate (ATP) production and consequently reducing gluconeogenesis which is a process that requires large amounts of energy to take place. Another proposed mechanism contributing to the reduction of gluconeogenesis is the alteration of the redox condition of the hepatocyte through the inhibition of the mitochondrial glycerophosphate dehydrogenase (mGPD) [41]. Furthermore, it is supposed that the energy imbalance produced by metformin could activate the 5′ adenosine monophosphate-activated protein kinase (AMPK) that would enhance catabolic pathways in order to restore the cellular energetic equilibrium [42]. More recently, an action of metformin also in the gut has been proposed, where it has shown to increase glucose uptake and use in the enterocytes and glucagon-like peptide-1 (GLP-1) secretion [43]. More specifically, the action of metformin in PCOS is supported by its insulin-sensitizing activity and its direct effect on androgen production. In fact, metformin has shown to enhance expression of GLUT4 mRNA in the adipose tissue of patients with PCOS [44] (which was found to be significantly lower compared to healthy controls).

The first trial assessing a beneficial role of metformin has been published in 1994 by Velazquez and Colleagues [45] who described a significant improvement in systolic blood pressure, insulin sensitivity, androgen, follicle-stimulating hormone (FSH), and SHBG levels after 8 weeks of therapy with metformin 1.5 g per day in 26 patients.

In a survey performed by the European Society of Endocrinology (ESE) PCOS Special Interest Group [46] on a group of experts mainly composed of endocrinologists, metformin was indicated as the respondent first choice for the overall treatment of PCOS.

However, a Cochrane meta-analysis [47] evaluating as secondary outcome fasting glucose levels has shown a reduction in fasting glucose with metformin compared to placebo or no treatments, with mean difference (MD −0.14 mmol/L; 95% CI −0.21 to −0.07).

On the contrary, a second meta-analysis performed by Patel and Shah [48] centred on metabolic outcomes of treatment with metformin versus placebo, synthetizing data on fasting blood glucose (FBG) levels of 13 randomized controlled trials (RCTs) and 506 subjects, reported no impact of metformin on fasting glucose levels (MD −1.38 mmol/L; 95% CI −3.71, 0.94).

Another Cochrane meta-analysis [49] compared metformin with oral contraceptive pill (OCP) and did not find significant differences for FBG. This meta-analysis was also inconclusive on the efficacy of metformin or the OCP alone or in combination, in the diabetes prevention, because of insufficient data.

When comparing metformin plus OCP to OCP alone, a better outcome on fasting glucose has been shown by Teede et al. [50] in favour of metformin plus OCP (MD 0.38 mmol/l; 95% CI0.22 to 0.54).

A trial performed in obese adolescents with PCOS and impaired glucose tolerance [51] evaluated three months of therapy with metformin and showed an improvement in glucose tolerance in this specific population; in fact, 8 out of 15 subjects had a normalization in their oral glucose tolerance test (OGTT) with significant lower posttreatment 2-hour glucose levels (9.1 ± 0.3 vs. 7.4 ± 0.3 mmol/l; *p* = 0.005).

In a retrospective study of 50 patients with PCOS [52], without diabetes at the baseline and treated with metformin, followed up for at least one year and with a mean follow-up of 40.2 months, researchers classified patients into two groups according to their baseline OGTT: those with (22%) and those without (78%) IGT. Only 5.1% of patients converted from normal glucose tolerance to IGT and 55% reversed from IGT to normal glucose tolerance. However, in the group of nonreversed IGT patients, there was an overall improvement of the OGTT which significantly decreased from 164 mg/dl to 135 mg/dl in the 2-hour plasma glucose level.

Metformin has no known adverse endometrial effects, or on the ovaries. Classical side effects associated with the treatment are gastrointestinal as diarrhoea, flatulence, anorexia, and abdominal discomfort. Lactic acidosis is an extremely rare complication and, in most cases, symptoms are usually mild and tend to reduce with habituation.

### 3.4. Metformin and Gestational Diabetes Mellitus

GDM is a glycemic alteration which can develop during pregnancy and, for physiopathological alterations, associated cardiovascular and metabolic risks are considered a model of the diabetic disease.

Women with PCOS have 2.8 to 4.3 times the risk of developing GDM during pregnancy, compared to women without PCOS [53–55]. This is because pregnancy condition is characterized by a series of metabolic changes promoting adipose tissue growth during the early phase, which is followed by an IR state with its peak during the 3^rd^ trimester [56, 57]. In women with an already IR state, addiction is another reason for IR and may lead to pregnancy complications. Evidence regarding the impact of metformin on the incidence of GDM is controversial [58–61]. A very recent meta-analysis evaluating the effect of metformin before conception and during pregnancy in women with PCOS and adjusting for study methodology, age, and BMI revealed that metformin treatment before or during pregnancy does not decrease the prevalence of GDM (*β* = −0.05, 95% CI −0.07, 0.04; *p* = 0.301) [62]. Another recently published RCT on 487 pregnant women comparing metformin treatment versus placebo, confirmed that metformin use does not prevent GDM (25% in the metformin group vs. 24% in the placebo group; OR 1.09, 95% CI 0.69–1.66; *p* = 0.75).

### 3.5. Thiazolidinediones

Through their action on the peroxisome proliferator-activated receptor gamma (PPAR-*γ*), thiazolidinediones (TZDs) are able to enhance the transcription of insulin sensitizer genes resulting in an increased expression of GLUT-4, lipoprotein lipase, fatty acid transporter protein, and fatty acyl CoA synthase. This results in a reduction of circulating fatty acids and increased synthesis of adiponectin favouring insulin-sensitizing effect and glucose uptake and lipogenesis in adipocytes.

TZDs have been less studied in PCOS, especially in the aspect regarding diabetes prevention and glucose tolerance deterioration.

Du et al. [63] performed a meta-analysis evaluating effects of rosiglitazone or pioglitazone versus placebo on metabolic outcomes. They selected six studies with FBG levels as outcome (including 219 patients: 105 receiving pioglitazone or rosiglitazone vs. 114 receiving placebo). With respect to the placebo, pioglitazone and rosiglitazone were more effective than placebo in reducing FBG levels (MD: −0.55 mmol/l; 95% CI −1.06 to −0.05; *p* = 0.031).

The same group performed another meta-analysis [64] including five trials comparing pioglitazone versus metformin on 258 patients, with fasting glucose levels as an outcome. Results showed a similar effect of the two drugs on FBG levels (MD: 0.21 mmol/l; 95% CI −0.46 to 0.03; *p* = 0.089).

In their study, Aroda and colleagues [65] compared 13 women with PCOS treated for 6 months with pioglitazone 45 mg per day with 10 women treated with placebo. They observed that patients treated with placebo had a deterioration of the glucose tolerance measured at the OGTT with respect to the group treated with placebo. Furthermore, in the placebo group, 29% of the patients with normal OGTT had an impaired glucose response at the end of the observation period, whereas none of the patients with impaired glucose tolerance response reverted to the normality. Meanwhile, in the pioglitazone arm, 40% of patients with altered OGTT reverted to normality and no one has experienced a worsening of their glucose tolerance status. With respect to metformin, thiazolidinediones have less gastrointestinal toxicities. A meta-analysis reported a statistically significant difference in adverse effects incidence between PCOS patients taking metformin and those in treatment with thiazolidinediones (OR = 8.88 for metformin vs. TZD, 95% CI = 3.54, 22.27; *p* < 0.00001) [66].

### 3.6. Sodium-Glucose Cotransporter-2 (SGLT2) Inhibitors

Since their effect on weight loss and IR, Javed et al. [67] suggested a potential role of SGLT2 inhibitors in the management of patients with PCOS. They performed aRCT, comparing hormonal and metabolic effect of empagliflozin 25 mg daily vs. metformin 1500 mg daily, treating 40 patients with PCOS and BMI over 25 kg/m^2^ for 12 weeks. May be because of the short period of observation or the small sample size, no changes in metabolic parameters (insulin, fasting glucose, and HOMA‐IR) have been observed from the baseline, nor on hormonal profile for both drugs. Only a favourable effect of empagliflozin on body weight (empagliflozin –1.4 kg ± 3.2% vs. metformin: 1.2 kg ± 2.3%; *p* = 0.006) and BMI (empagliflozin: −1.4 ± 3.2% vs. metformin: 1.1 ± 2.2%; *p* = 0.006) has been observed.

### 3.7. GLP-1 Receptor Agonist and DPP4 Inhibitors

Glucagon-like peptide-1 receptor agonists (GLP-1 RA) and dipeptidyl peptidase-4 inhibitors (DPP4i) are new molecules utilized for the treatment of T2D which are able to increase insulin secretion by miming incretins action or prolonging endogenous hormone half-life.

Liraglutide has been approved in different countries for obesity treatment at doses higher than those used for T2D. This marked effect on anthropometric parameters could be beneficial in PCOS women, where a prevalence of obese patients ranging from 50 to 80% has been estimated. In this perspective, some studies have been performed, evaluating effect of this molecule in PCOS.

Assuming a synergist effect of liraglutide and metformin on insulin and IR, Jensterle et al. [68] evaluated 30 obese women with PCOS, randomized to be treated with the combination of metformin 1000 mg twice a day and liraglutide 1.2 mg once a day or liraglutide alone 3 mg once a day. After 12 weeks of treatment, the authors observed a significant improvement in the OGTT at 0 and 120 min in the combination therapy arm (*p* = 0.015).

Saxagliptin is a selective oral dipeptidyl peptidase-4 (DPP4) inhibitor. A recent study [69] evaluated metabolic parameters of 75 patients diagnosed with both T2D and PCOS randomly assigned to treatment with metformin (2000 mg daily) or saxagliptin (5 mg daily) or both, in over 24 weeks. The study showed significant reduction of glycated haemoglobin in all groups, but no differences were detected between groups (saxagliptin vs. combination vs. metformin: −1.1% vs. −1.3% vs. −1.1%, respectively, *p* = 0.016; saxagliptin vs. metformin, *p* = 0.890).

Another study [69] performed on premenopausal patients with PCOS and impaired fasting glucose or impaired glucose tolerance randomly assigned patients to three different treatments: metformin extended release 2000 mg once daily, saxagliptin 5 mg once daily, and the combination of both 5 mg saxagliptin and 2000 mg metformin once daily. At the end of the study, 56% of patients had a glucose tolerance normalization (25% in the metformin group, 55% in the saxagliptin, and 91% in the combination). The arm with saxagliptin plus metformin demonstrated superiority versus metformin in fasting glucose and OGTT normalization (*p* = 0.007). Most commonly reported side effect of these classes of drugs is nausea; however, the overall tolerability is good.

### 3.8. Inositol

Inositol is a cyclitol present in animal and plant cells. It has nine distinct stereoisomers, myo-inositol being the most widespread. D-Chiro-inositol is an inositol isoform derived from myo-inositol through an epimerization process [70]. Both myo- and d-chiro-inositol showed insulin mimetic effects in animal models of IR [71]. The physiological myo-inositol : d-chiro-inositol ratio and their relative concentration are tissue-specific reflecting their different functions. Indeed, myo-inositol increases glucose cellular uptake and plays a role in the FSH activity, while d-chiro-inositol is crucial for glycogen synthesis and is involved in the insulin-induced overproduction of androgens in the ovary.

Inositol has been mainly used as a supplement in treating several pathologies such as metabolic syndrome [72, 73], GDM [74–81], T2D [82], and PCOS [83, 84].

In postmenopausal women affected by metabolic syndrome, myo-inositol administration, in addition to diet, resulted in significantly reducing several parameters, in comparison with patients only on diet. Myo-inositol decreased diastolic blood pressure (−11%), HOMA index (−75%), and serum triglycerides (−20%); furthermore, the treatment increased high-density lipoprotein cholesterol (+22%). The authors stated that myo-inositol administration may be a therapeutic opportunity for treating metabolic syndrome in postmenopausal women [72].

In the last years, several clinical studies demonstrated the significant efficacy of inositols (mainly myo-inositol) in GDM prevention [76–80]. The largest benefit in the myo-inositol group compared to d-chiro-inositol was clearly demonstrated. The relevance of these findings is mainly related to the possibility of an effective therapeutic approach in GDM using myo-inositol alone at a dosage of 4000 mg daily, which gave a lower relative risk for an abnormal maternal OGTT [80]. Also, a very recent systematic review and meta-analysis [81] of five RCTs (965 participants) significantly supported the therapy with myo-inositol alone or plus d-chiro-inositol in the prevention of GDM. To date, only a pilot study, relevant for possible clinical implications in the future, evaluated the administration of inositol in 20 subjects affected by T2D, with suboptimal glycemic control already treated with glucose-lowering agents. Patients (five males and fifteen females) were treated orally for three months with myo-inositol plus d-chiro-inositol in the 40 : 1 ratio as add-on supplement to their glucose-lowering drugs. After three months of treatment, fasting blood glucose and HbA1c levels significantly decreased compared to the baseline. There was no significant difference in blood pressure, lipid profile, and BMI levels [82].

Three reviews including a total of 62 trials with 5072 women with PCOS evaluated the efficacy of inositol in PCOS [47, 85, 86]. Endocrine outcomes were assessed by all the reviews with benefits reported in testosterone, dehydroepiandrosterone (DHEA), or androstenedione levels. Metabolic outcomes were assessed by all the reviews with important results: reduced insulin levels [47, 72, 85], reduced glucose levels and HOMA-IR, and improved blood pressure, triglycerides, and high-density lipoprotein [86]. The effectiveness of myo-inositol for PCOS was investigated by a systematic review and meta-analysis of RCTs [87]. Authors showed that myo-inositol is likely to improve IR because of the lower HOMA index both in the myo-inositol vs. placebo comparison (WMD −1.11; 95% CI −1.39–0.83; *p* < 0.00001) and in the myo-inositol vs. metformin or estrogen comparison (WMD −0.33; 95% CI −0.60–0.05; *p* = 0.02). In the myo-inositol vs. placebo subgroup, myo-inositol is likely to improve hyperinsulinemia because of the lower fasting insulin (WMD −3.97; 95% CI −5.76, −2.18; *p* < 0.00001), while in the myo-inositol vs. metformin or estrogen subgroup, there is not enough strong evidence (WMD −0.99; 95% CI −2.43, 0.45; *p* = 0.19). In each subgroup, there is not enough strong evidence that myo-inositol has an effect on fasting glucose.

Another meta-analysis analysed 9 RCTs involving 247 cases and 249 controls. The results of quantitative analyses showed a significant decrease of fasting insulin (SMD = −1.021 *μ*U/mL, 95% CI: −1.791 to −0.251, *p* = 0.009) and homeostasis model assessment (HOMA) index (SMD = −0.585, 95% CI: −1.145 to −0.025, *p* = 0.041) in the group treated with myo-inositol. A meta-analysis comparing myo-inositol vs. metformin in PCOS women showed no difference in the short-term effect for fasting insulin, HOMA index, testosterone, androstenedione, SHBG, and BMI, further highlighting the absence of side effects in the myo-inositol group compared to the metformin one [88]. Indeed, inositol is generally well tolerated on the dosage regimen commonly used, and it has extensively proved to improve the metabolic profile of PCOS women, along with a reduction of their hyperandrogenism.

Further studies have also shown the potential of inositols to restore spontaneous ovulation and improve fertility in women with PCOS [83].

An important concept in treatment with inositols is the balance between the two isoforms myo-inositol and d-chiro-inositol. D-Chiro-inositol is in fact a derivative of myo-inositol, produced by the action of an epimerase which is regulated by insulin. Muscle, fat, and liver are characterized by higher concentrations of d-chiro-inositol, while those of the heart and brain are lower. In PCOS, insulin resistance produces an imbalance of the myo-inositol : d-chiro-inositol ratio, this is because of the overstimulation of the epimerase due to the hyperinsulinemic state. Interestingly, the ratio 40 : 1 of myo-inositol/d-chiro-inositol seems to be the most appropriated clinical approach for overweight/obese PCOS women and, in a study exploring different isomers ratios (1 : 3.5; 2.5 : 1; 5 : 1; 20 : 1; 40 : 1; 80 : 1), the 40 : 1 myo-inositol/d-chiro-inositol has shown to better restore ovulation and improve hormonal levels, basal and postprandial insulin levels, and HOMA index [89]. Indeed, administration of myo-inositol plus d-chiro-inositol in a ratio 40 : 1 reduced the metabolic and clinical alteration of PCOS, therefore reducing the risk of developing metabolic syndrome [90, 91].

### 3.9. Limitations

In the majority of studies in PCOS treatment primary objectives of the trials are changes from the baseline in hormonal parameters or ovulatory function resumption or improvement in menstrual irregularities; thus, studies results were underpowered to detect changes in metabolic parameters or in diabetes prevention when these are considered as secondary outcomes. Indeed, very few studies analyse prediabetes treatment in patients with PCOS and some of them are retrospective. Furthermore, a majority of them have been designed with a small sample size and the observation time is often very short; it does not exceed 24 weeks for longer studies.

## 4. Conclusions

T2D and PCOS are both common conditions associated with IR and compensatory hyperinsulinemia. It is acknowledged that an impaired glucose tolerance is common in women with PCOS and a higher risk of developing polycystic ovaries has been found in women with T2D compared to the general population. Therefore, does treating one condition treat the other? Improvement of insulin sensitivity can normalize endocrine and reproductive disorders. Clinical evidence has shown how the use of insulin-sensitizing agents, such as metformin and inositols, may improve the endocrine and metabolic profile in PCOS women.

One study suggested a possible effect of myo-inositol in the primary prevention of GDM in PCOS women; however, studies analysing preventive effects from T2D in PCOS women are still lacking. The higher tolerability of myo-inositol, compared to metformin, may improve the patient compliance and therefore be more suitable for a longer administration period. Treatment with liraglutide could be beneficial, particularly in obese women. The efficacy of SGLT2i should be tested in better designed studies. New studies addressing different PCOS phenotypes and possible association with metabolic disorders are recommended. Long-term clinical controlled trials, using insulin-sensitizing agents along with lifestyle interventions, are needed in order to further prove their effectiveness in reducing the incidence of T2D and improving the metabolic and endocrine profile in women with PCOS.

## Figures and Tables

**Table 1:** Effect of different treatments on impaired glucose tolerance and impaired fasting glucose in women with polycystic ovary syndrome.

Intervention	Impaired glucose tolerance (IGT)	Impaired fasting glucose (IFG)
Lifestyle modifications	Improved (28, 29, 33, 34)	Improved (28, 29, 33, 34)
Metformin	(i) Improvement in 2-hour glucose levels at the OGTT (46, 47)	Reduction in fasting glucose levels compared to placebo (42)
	(ii) Reversion from IGT to normal glucose tolerance (47)	
Thiazolidinediones	Reversion from IGT to normal glucose tolerance (60)	Reduction in fasting glucose levels compared to placebo (58)
SGLT2i	Neutral effect (61)	Neutral effect (61)
GLP-1 RA	Improved with the combination of liraglutide and metformin (62)	Improved with the combination of liraglutide and metformin (62)
DPP4i	Improved with the combination of saxagliptin and metformin (63)	Improved with the combination of saxagliptin and metformin (63)
Inositol	Improved (78)	Improved (78)

SGLT2i, sodium-glucose cotransporter-2 inhibitors; GLP-1 RA, glucagon-like peptide-1 receptor agonists; DPP4i, dipeptidyl peptidase-4 inhibitors; OGTT, oral glucose tolerance test.
